# Predicting Venous Thrombosis in Osteoarthritis Using a Machine Learning Algorithm: A Population-Based Cohort Study

**DOI:** 10.3390/jpm12010114

**Published:** 2022-01-15

**Authors:** Chao Lu, Jiayin Song, Hui Li, Wenxing Yu, Yangquan Hao, Ke Xu, Peng Xu

**Affiliations:** 1 Department of Joint Surgery, Xi’an Hong Hui Hospital, Xi’an Jiaotong University Health Science Center, Xi’an 710054, China; luchao0925@163.com (C.L.); jean_songdm@sina.com (J.S.); lihui1326148739@163.com (H.L.); yuwenxing110@163.com (W.Y.); haoyq2008@126.com (Y.H.); 2 Department of Traditional Chinese and Western Medicine, The First Clinical College of Shaanxi University of Chinese Medicine, Shaanxi University of Chinese Medicine, Xi’an 712046, China

**Keywords:** osteoarthritis, venous thrombosis, VTE risk prediction, machine learning algorithm, population-based cohort study

## Abstract

Osteoarthritis (OA) is the most common joint disease associated with pain and disability. OA patients are at a high risk for venous thrombosis (VTE). Here, we developed an interpretable machine learning (ML)-based model to predict VTE risk in patients with OA. To establish a prediction model, we used six ML algorithms, of which 35 variables were employed. Recursive feature elimination (RFE) was used to screen the most related clinical variables associated with VTE. SHapley additive exPlanations (SHAP) were applied to interpret the ML mode and determine the importance of the selected features. Overall, 3169 patients with OA (average age: 66.52 ± 7.28 years) were recruited from Xi’an Honghui Hospital. Of these, 352 and 2817 patients were diagnosed with and without VTE, respectively. The XGBoost algorithm showed the best performance. According to the RFE algorithms, 15 variables were retained for further modeling with the XGBoost algorithm. The top three predictors were Kellgren–Lawrence grade, age, and hypertension. Our study showed that the XGBoost model with 15 variables has a high potential to predict VTE risk in patients with OA.

## 1. Introduction

Osteoarthritis (OA) is the most common joint disease worldwide, with an age-associated increase in both incidence and prevalence [1,2]. It is estimated that approximately 302 million people globally suffer from this disease, and the associated healthcare resources and financial burden can be substantial [3,4]. OA, a primary cause of pain, disability, and joint replacement, is characterized by disease affecting the whole joint, including articular cartilage degradation, synovium and ligament inflammation, and changes to the subchondral bone [5,6,7]. Despite the symptomatic treatment of pain, stiffness, and swelling, there are no FDA-approved disease-modifying drugs [8]. As a complex disease, a multitude of possible etiologies contribute to the development of OA, including obesity, sedentary lifestyle, trauma, and aging [9,10,11]. Early prevention and elimination of risk factors are critical in delaying disease progression [12]. Nevertheless, despite these identifiable underlying causes, OA still cannot be effectively prevented.

Venous thrombosis is a relatively common and potentially fatal condition in patients, and an increased risk of VTE has been reported in arthritis, particularly in rheumatic arthritis (RA) [13,14,15,16]. Li et al. reported that RA patients have an increased risk of VTE, pulmonary embolism, and deep vein thrombosis after diagnosis in comparison with the general population [17]. This suggests that VTE may play a vital role in chronic and systemic inflammatory autoimmune disease. However, the relationship between OA and VTE has not been elucidated. A recent study in a large population-based cohort revealed that knee or hip osteoarthritis might increase incident VTE risk to 40% and 80%, respectively, when compared to those without OA, which may be partly mediated through joint replacement [18].

Thus, predicting the VTE risk among OA patients is critical to reduce morbidity and mortality from VTE in OA patients. Machine learning (ML) is a computer-based method of data analysis that is often used to construct predictive models based on large datasets [19]. In this study, we aimed to develop a model using the ML algorithm to identify those at high risk of VTE in OA patients

## 2. Materials and Methods

We performed a single-center cross-sectional study of OA patients in Xi’an Honghui Hospital between January 2018 and December 2020. Patients were consecutively recruited from joint surgery department and were examined by venous ultrasound of the legs to assess VTE risk. The inclusion criteria were as follows: (1) diagnosed with knee osteoarthritis (guidelines for the diagnosis and treatment of osteoarthritis (2018 edition)) [20]; (2) radiographically evaluated by X-ray at Kellgren–Lawrence grade stages 3–4. Those with heart stent, ischemic stroke, cancers, or incomplete laboratory data were excluded from the study. The study was approved by the Ethics Committee of Xi’an Honghui Hospital and conducted in accordance with the Declaration of Helsinki. Written informed consent was waived owing to the retrospective nature of the study. All confidential patient information was deleted from the entire dataset prior to the analysis.

All patient demographics and laboratory data at admission were extracted manually from electronic medical records using a standardized case report form.

### 2.1. Machine Learning Algorithms

To develop machine learning models, 35 parameters were used for the analysis. Before developing the ML models, laboratory indices, which were continuous variables, were converted into categorical variables based on their normal range values. In addition, the patient’s age was treated as a continuous variable, with missing values replaced by median values. All patients were randomly divided into a training set and test set at a ratio of 8:2.

Six ML algorithms, namely logistic regression (LR), random forest (RF), extreme gradient boosting (XGBoost), adaptive boosting (AdaBoost), gradient boosting decision tree (GBDT), and light gradient boosting machine (LGBM), were used to predict the VTE risk. We used the receiver operating characteristic (ROC) curve as the evaluation metric to compare the performance of the ML algorithm between the training and testing sets. The best performance model was chosen, and recursive feature elimination (RFE) was employed to screen the optimized variable combinations. For model interpretation, the Shapley additive exPlanations (SHAP) algorithm was used to calculate the Shapley value of each variable based on game theory to further explain the best performance model.

### 2.2. Statistical Analysis

All statistical analyses were conducted using Python software (version 3.8). A Fisher’s exact test or an x^2^ test was conducted for binary variables, and Student’s *t*-test was used for continuous variables. Owing to the imbalance of the dataset, the synthetic minority oversampling technique (SMOTE) was used to deal with the training set. Six ML algorithms were used to screen for the best performance prediction model. Using the RFE algorithm, all variables were filtered one by one to obtain the best combination, which was then established in a selected ML prediction model. We also used the SHAP algorithm to interpret and evaluate the optimized model. Statistical significance was set at *p* ≤ 0.05.

## 3. Results

**Figure 1:**
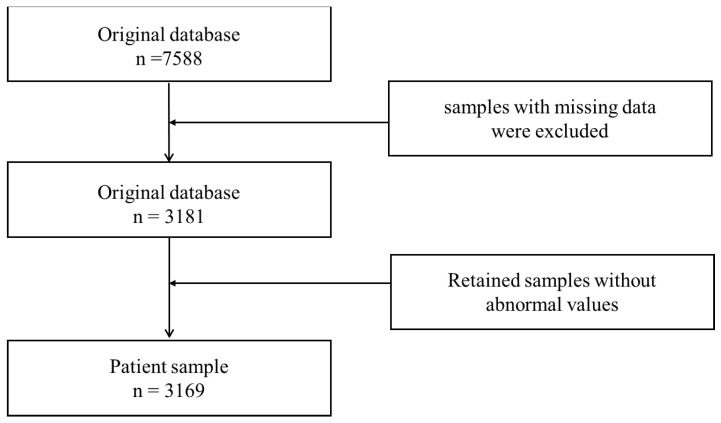
Flow chart of patients for enrollment.

We excluded subjects with missing data and subsequently enrolled 3169 patients with an average age of 66.52 ± 7.28 years in the study (Figure 1). Of them, 2400 patients were male and 769 patients were female, accounting for 75.73% and 24.27% of all patients, respectively. All patients were divided into the VTE and non-VTE groups. There were 352 patients with VTE, with an average age of 68.05 ± 6.84 and 2817 patients without VTE, with an average age of 66.33 ± 7.31. In the VTE group, 281 patients were male (79.83%) and 71 patients were female (20.17%). In the non-VTE group, 2119 patients were male (75.22%) and 698 were female (24.78%). The baseline characteristics of patients stratified by VTE are summarized in Table 1.

**Figure 2:**
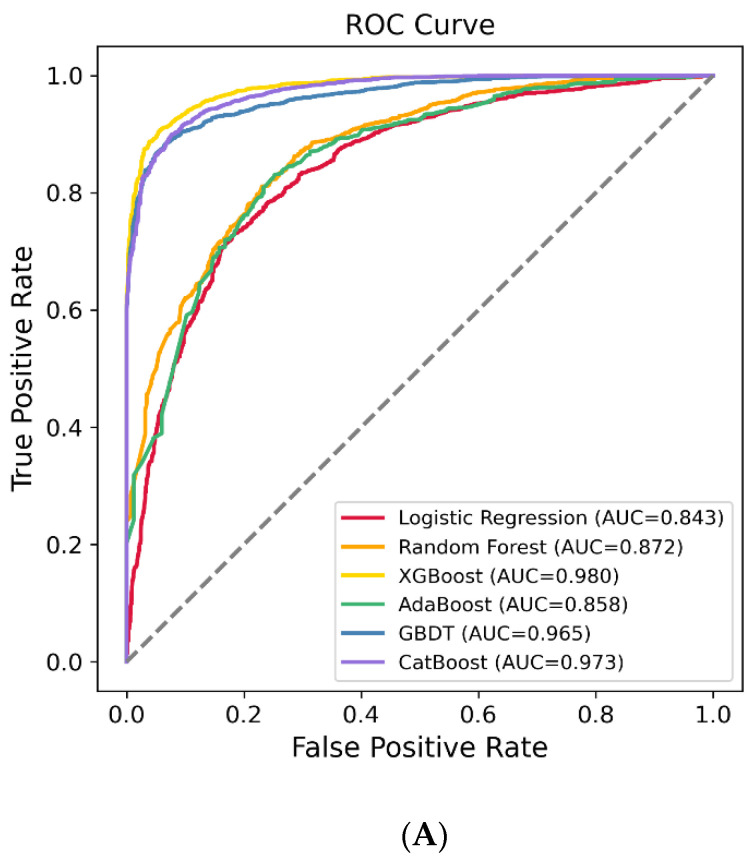

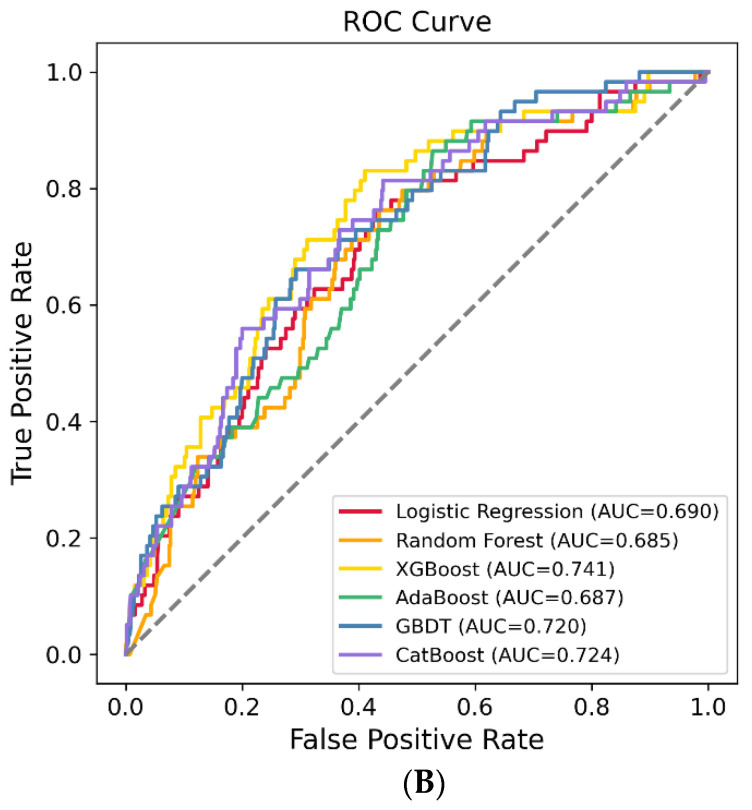
The receiver operating characteristic (ROC) curves of the machine learning models on the training set (**A**) and testing set (**B**).

The patients were randomly stratified (8:2) into training and testing sets to evaluate the model performance. Finally, a total of 35 characteristics were enrolled in the six ML algorithms, including LR, RF, XGBoost, AdaBoost, GBDT, and LGBM, to identify the model with the best predictive performance. Our results showed that the XGBoost model demonstrated the best performance, with an area under the curve (AUC) of 0.741 (95% CI: 0.676, 0.806) (Figure 2A,B). The AUC values of the other models are shown in Table 2.

**Figure 3:**
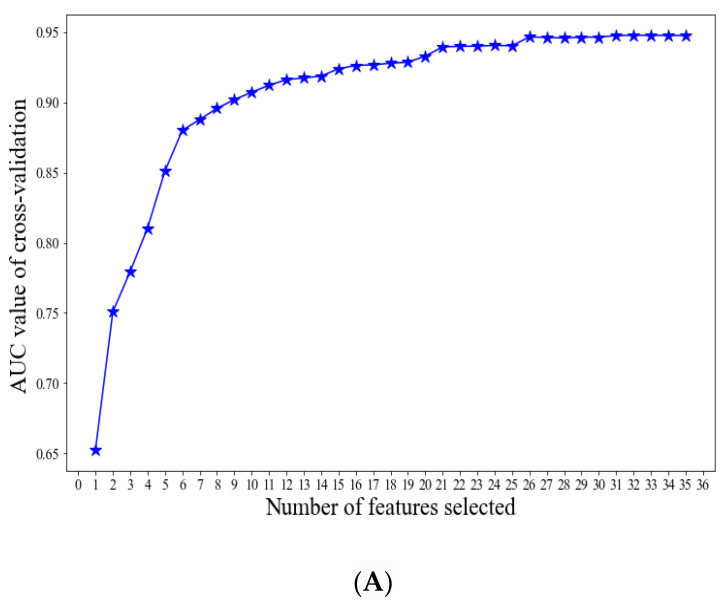

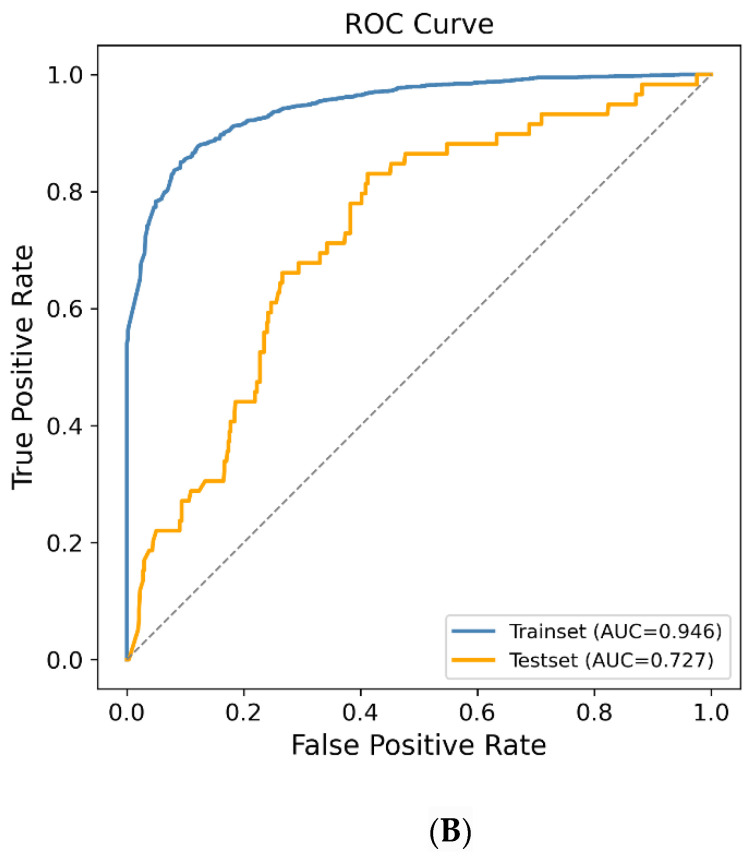
Using the RFE method to screen the optimal variables. (**A**) The most import variables, screened by the RFE method; (**B**) The receiver operating characteristic (ROC) curves of XGBoost model on the training set and testing set.

To further optimize the XGBoost model, the RFE method was used to screen the most important variables that can predict the VTE risk. Finally, 15 variables were employed to establish the final prediction model, and the new XGBoost model showed that the AUC of the testing dataset was 0.727 (95% CI = 0.662, 0.792) (Figure 3A,B).

### Interpretation and Evaluation of Machine Learning Model

**Figure 4:**
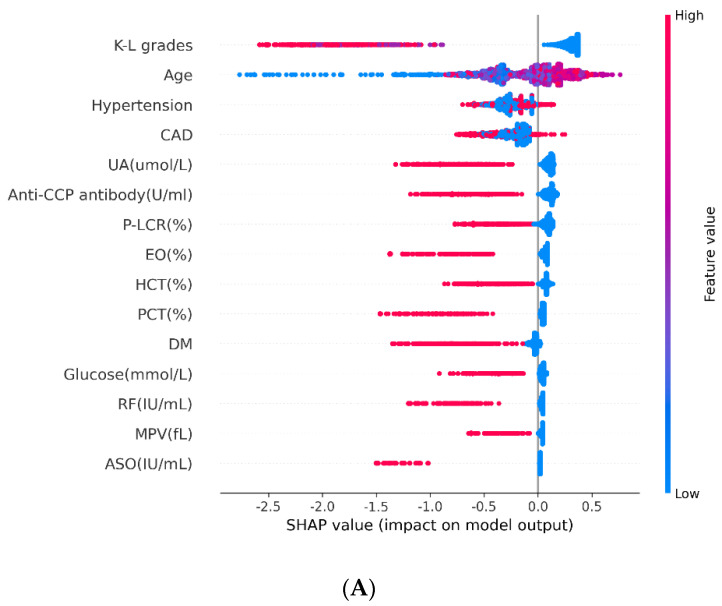

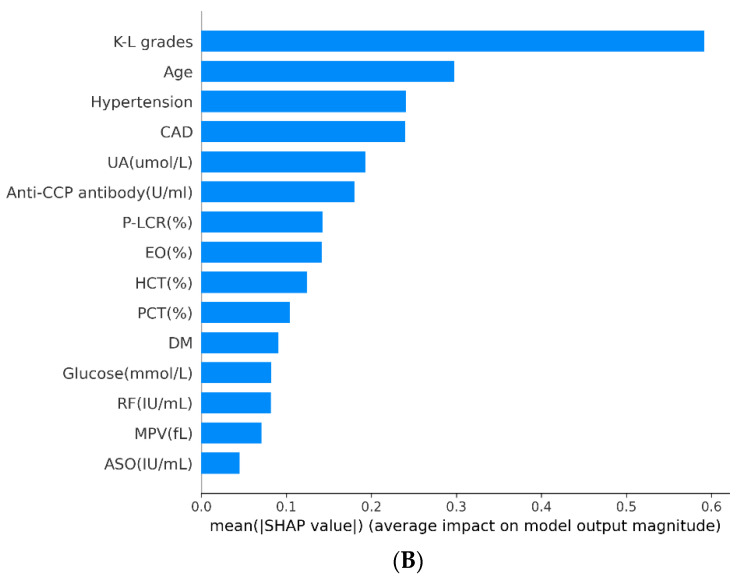
Interpretation and Evaluation of Machine Learning Model. (**A**) SHAP analysis on the dataset, which shows the 15 most important features and their impact on the model output. Each dot represents one patient, with blue color meaning the lowest range and red color meaning the highest range of the feature; (**B**) Ranking of the features’ importance indicated by SHAP analysis.

The SHAP method was also used to interpret the relative importance of each variable in the XGBoost model. Our results showed that age, eosinophil ratio (EOSR), hematocrit (HCT), mean platelet volume (MPV), thrombocytocrit (PCT), platelet-larger cell ratio (P-LCR), uric acid (UA), glucose, antistreptococcal hemolysin “O” (ASO), anti-cyclic citrullinated peptide antibody (ACPA), rheumatoid factor (RF), Kellgren–Lawrence grade (K–L grade), history of hypertension, diabetes, and coronary artery disease (CAD) were associated with the risk of VTE in OA patients. Particularly, K–L grade, age, and hypertension were the three vital variables (Figure 4A,B).

## 4. Discussion

Extensive efforts have been made to delay OA patients progress to the end stage. In this hospital-based cross-sectional study, we used the ML algorithm to predict VTE risk in patients with OA. We found that using the XGBoost model with 15 variables can predict VTE risk in OA patients, and this may have a growing prevalence due to the global ageing population.

OA is not simply a matter of mechanical damage to the joint but involves several additional risk factors [21]. Nevertheless, some patients still inevitably rapidly progress to the end stages [22]. The 11th leading cause of disability worldwide has resulted in a rapid increase in orthopedic surgeries over the last few decades [4]. Rather than medication, lifestyle modification is the most promising avenue for the prevention of OA [3,23]. Many risk factors, including VTE, have been identified, and these may be partly mediated through knee or hip replacement. In a large population-based cohort study, Zeng et al. reported that VTE increased by approximately 40% among individuals with knee OA and by 80% among individuals with hip OA compared to those without OA [18].

Machine learning is a crucial branch of artificial intelligence that utilizes historical data to predict the likelihood of a future outcome [24,25]. As a multidisciplinary approach, ML algorithms are increasingly being utilized to predict outcomes in lower-extremity total joint arthroplasty [26]. Lu et al. used ML to establish a model to predict surgical outcomes after non-compartmental knee arthroplasty [27]. Kunze et al. developed machine learning algorithms based on partially modifiable risk factors for predicting dissatisfaction after arthroplasty [28]. In this study, we found that the XGBoost algorithm was the best performing algorithm. In this prediction model, 15 variables were found to be associated with VTE risk. In addition to the conventional risk factors such as age, hypertension, and diabetes, our study found that CAD, EOSR, HCT, MPV, PCT, P-LCR, UA, ASO, ACPA, RF, and Kellgren–Lawrence grade were also correlated with VTE. These have not been reported elsewhere.

The present study has certain limitations. First, although ML algorithms are widely used in medical practice, the predictive value is limited due to the “black box” characteristic. Thus, rather than being used as a clinical judgment tool, an ML algorithm model should be used as a reference for physicians. Second, all the data analyzed in the present study were from a single institution, and the imbalance of gender ratio has limited the generalization of our results. Additionally, because of the nature of an observational study, some unmeasured confounding effects may persist; thus, additional validation and assessment of the relationship between the variables and VTE in OA patients should be performed in a large population. Nevertheless, despite such limitations, to our knowledge, this is the first study to use a machine learning method to predict VTE risk in OA patients.

## 5. Conclusions

In conclusion, we developed a XGBoost model with a high accuracy in the prediction of VTE risk in patients with OA, which might supply a complementary tool for the screening of populations at high risk of VTE.

## Figures and Tables

**Table 1:** Characteristics of the patients stratified by VTE or not.

	Class ^a^	Total	None-Venous Thrombosis	Venous Thrombosis	*p* ^b^
N		3169	2817	352	
Age (year) ^b^		66.52 ± 7.28	66.33 ± 7.31	68.05 ± 6.84	<0.001
Gender					
	Male	2400 (75.73%)	2119 (75.22%)	281 (79.83%)	0.066
	Female	769 (24.27%)	698 (24.78%)	71 (20.17%)	
Hypertension					
	No	1730 (54.59%)	1543 (54.77%)	187 (53.12%)	0.597
	Yes	1439 (45.41%)	1274 (45.23%)	165 (46.88%)	
Diabetes					
	No	2751 (86.81%)	2437 (86.51%)	314 (89.20%)	0.185
	Yes	418 (13.19%)	380 (13.49%)	38 (10.80%)	
Coronary heart disease					
	No	2207 (69.64%)	1974 (70.07%)	233 (66.19%)	0.152
	Yes	962 (30.36%)	843 (29.93%)	119 (33.81%)	
Kellgren–Lawrence grade					
	0	2269 (71.60%)	1943 (68.97%)	326 (92.61%)	<0.001
	III	181 (5.71%)	178 (6.32%)	3 (0.85%)	
	IV	719 (22.69%)	696 (24.71%)	23 (6.54%)	
Eosinophil ratio					
	Normal Range	2746 (86.65%)	2431 (86.30%)	315 (89.49%)	0.115
	Abnormal	423 (13.35%)	386 (13.70%)	37 (10.51%)	
Hematocrit					
	Normal Range	2535 (79.99%)	2254 (80.01%)	281 (79.83%)	0.991
	Abnormal	634 (20.01%)	563 (19.99%)	71 (20.17%)	
Mean platelet volume					
	Normal Range	2782 (87.79%)	2462 (87.40%)	320 (90.91%)	0.070
	Abnormal	387 (12.21%)	355 (12.60%)	32 (9.09%)	
Thrombocytocrit					
	Normal Range	2858 (90.19%)	2527 (89.71%)	331 (94.03%)	0.013
	Abnormal	311 (9.81%)	290 (10.29%)	21 (5.97%)	
platelet-larger cell ratio					
	Normal Range	2390 (75.42%)	2112 (74.97%)	278 (78.98%)	0.114
	Abnormal	779 (24.58%)	705 (25.03%)	74 (21.02%)	
Uric acid					
	Normal Range	2554 (80.59%)	2261 (80.26%)	293 (83.24%)	0.208
	Abnormal	615 (19.41%)	556 (19.74%)	59 (16.76%)	
Glucose					
	Normal Range	2665 (84.10%)	2369 (84.10%)	296 (84.09%)	0.941
	Abnormal	504 (15.90%)	448 (15.90%)	56 (15.91%)	
Antistreptococcal hemolysin “O”					
	Normal Range	3074 (97.00%)	2726 (96.77%)	348 (98.86%)	0.045
	Abnormal	95 (3.00%)	91 (3.23%)	4 (1.14%)	
Anti-CCP antibody					
	Normal Range	2549 (80.44%)	2255 (80.05%)	294 (83.52%)	0.140
	Abnormal	620 (19.56%)	562 (19.95%)	58 (16.48%)	
Rheumatoid factors					
	Normal Range	2902 (91.57%)	2577 (91.48%)	325 (92.33%)	0.661
	Abnormal	267 (8.43%)	240 (8.52%)	27 (7.67%)	

^a^ Continuous variable are transformed to dichotomous variables according to their normal range. ^b^ Values are presented as mean ± SD.

**Table 2:** The area under the curve (AUC) of training set and testing set.

	Training Set (AUC, 95% CI)	Testing Set (AUC, 95% CI)
LR	0.843 (0.832, 0.855)	0.690 (0.620, 0.760)
RF	0.872 (0.862, 0.882)	0.685 (0.618, 0.753)
XGBoost	0.980 (0.977, 0.983)	0.741 (0.676, 0.806)
AdaBoost	0.858 (0.847, 0.868)	0.687 (0.619, 0.755)
GBDT	0.965 (0.960, 0.970)	0.720 (0.656, 0.784)
CatBoost	0.973 (0.969, 0.977)	0.724 (0.657, 0.790)

## Data Availability

The authors confirm that all data underlying the findings are fully available and can be obtained after submitting a request to the corresponding author.
